# Antibacterial and Antibiofilm Properties of Three Resin-Based Dental Composites against *Streptococcus mutans*

**DOI:** 10.3390/ma15051891

**Published:** 2022-03-03

**Authors:** Simonetta D’Ercole, Francesco De Angelis, Virginia Biferi, Chiara Noviello, Domenico Tripodi, Silvia Di Lodovico, Luigina Cellini, Camillo D’Arcangelo

**Affiliations:** 1 Department of Medical, Oral and Biotechnological Sciences, “G. d’Annunzio” University of Chieti–Pescara, 66100 Chieti, Italy; simonetta.dercole@unich.it (S.D.); virginia.biferi@unich.it (V.B.); chiara96sgr@gmail.com (C.N.); tripodi@unich.it (D.T.); cdarcang@unich.it (C.D.); 2 Department of Pharmacy, “G. d’Annunzio” University of Chieti–Pescara, 66100 Chieti, Italy; silvia.dilodovico@unich.it (S.D.L.); l.cellini@unich.it (L.C.)

**Keywords:** composite resins, *Streptococcus mutans*, biofilm formation, dental caries, bacterial adhesion

## Abstract

Antibacterial and antibiofilm properties of restorative dental materials may improve restorative treatment outcomes. The aim of this in vitro study was to evaluate *Streptococcus mutans* capability to adhere and form biofilm on the surface of three commercially available composite resins (CRs) with different chemical compositions: GrandioSO (VOCO), Venus Diamond (VD), and Clearfil Majesty (ES-2). Disk-shaped specimens were manufactured by light-curing the CRs through two glass slides to maintain a perfectly standardized surface topography. Specimens were subjected to Planktonic OD_600nm_, Planktonic CFU count, Planktonic MTT, Planktonic live/dead, Adherent Bacteria CFU count, Biomass Quantification OD_570nm_, Adherent Bacteria MTT, Concanavalin A, and Scanning Electron Microscope analysis. In presence of VOCO, VD, and ES2, both Planktonic CFU count and Planktonic OD_600nm_ were significantly reduced compared to that of control. The amount of Adherent CFUs, biofilm Biomass, metabolic activity, and extracellular polymeric substances were significantly reduced in VOCO, compared to those of ES2 and VD. Results demonstrated that in presence of the same surface properties, chemical composition might significantly influence the in vitro bacterial adhesion/proliferation on resin composites. Additional studies seem necessary to confirm the present results.

## 1. Introduction

The use of composite resin (CR)-based materials in restorative dentistry considerably grew in recent years, mainly due to their latest improvements in mechanical properties and esthetic features [1,2,3,4]. CRs can be successfully employed for small- and medium-sized adhesive restorations, with direct techniques, and even when dealing with wide restorations and extensive rehabilitations, following an indirect approach [5,6]. However, oral cavities may present many challenges to their physical and chemical stability, mainly due to an extremely moist environment and the frequent temperature/pH variations [7]. Acids released by acidogenic bacteria such as *Streptococcus mutans*, together with the degradative attack pursued by enzymes present in saliva, can progressively compromise the CR functional and esthetic features over time, which may even result in interfacial gaps at the margins of restoration [8].

Many research efforts into studying the bacterial biofilm formation on composite resins were conducted. Biofilms adhering to CRs contain microorganisms, such as streptococci and lactobacilli, that can survive in a highly acidic environment [9]. The amount of *S. mutans* in biofilm is particularly relevant as it plays a key role in determining its cariogenic potential [10]. Many studies showed that CR surface is susceptible to bacterial adhesion, mainly in the marginal areas of restorations [11,12]. Moreover, the adhesion phase is strongly influenced by surface characteristics such as surface roughness (Ra) and surface free energy [13]. An Ra value below 0.2 μm is considered to have a low effect on bacterial adhesion in vivo [14]. Thus, optimizing surface properties of dental composite resins through an accurate finishing and polishing can contribute to restoration success by preventing biofilm formation [15,16,17].

Beyond surface roughness, other parameters seem to be able to significantly affect bacterial adhesion on composite resins. Aside from the *ad hoc* inclusion of antibacterial additives, it could be interesting to better investigate if even for general composites, the inherent CR formulation could play a relevant role in biofilm formation phases [18,19]. For instance, there are controversial results in literature concerning the correlation between biofilm formation and filler particle size [18,20,21]: several studies reported that microhybrid composites might lead to higher bacterial adhesion than nanohybrids [22], while other studies observed no differences [21]. Likewise, concerning the effect of the organic matrix components on bacteria, a study by Van Landuyt et al. showed that in presence of ethylene glycol dimethacrylate (DEGDMA), triethylene glycol dimethacrylate (TEGDMA), or diethylene glycol dimethacrylate (EGDMA), the count of *Streptococcus sanguinis* and *Streptococcus sobrinus* were similarly decreased compared to that of the broth control after 6 h and 9 h incubation periods, respectively, with no significant differences among the tested monomers [23]. As well, Takahashi et al. reported no differences between the effect of TEGDMA and DEGDMA on CFU numbers (CFU/mL) of *S. sobrinus* and *S. sanguinis* [24]. On the other hand, other results suggest that different monomers might differently affect cell proliferation [25,26].

Based on the above-mentioned contrasting findings, the aim of the present in vitro study was to analyze the *S. mutans* capability to adhere and form biofilm on the surface of three commercially available CRs with different chemical compositions. The null hypothesis was that there are no differences in terms of antibacterial and antibiofilm properties among chemically different resin composites if they show the same standardized surface topography.

## 2. Materials and Methods

The list of the composite resins included in the experimental design, together with their composition, is given in Table 1.

### 2.1. Realization of Composite Disks

Disk-shaped specimens were prepared by positioning the uncured material in a polyvinylsiloxane mold, with a diameter of 4 mm and a height of 2 mm, resulting in a total surface area of 50.27 mm^2^. To achieve a perfectly smooth and standardized surface topography, the samples were inserted between two glass slides and stuck with a paper clip for 20 s to extrude the excess material. They were then light-cured for 20 s from the upper surface and for 20 s from the lower surface, using a light-emitting-diode curing unit (Celalux 3, VOCO, Cuxhaven, Germany) with 8 mm diameter tip and an output power of 1300 mW/cm^2^. All disks were washed in an ultrasonic bath and were not subjected to any further surface treatment.

### 2.2. Saliva Collection

Human saliva samples were taken from healthy volunteers with age >18 years, according to a previously described protocol. The Ethics Committee of University “G. d’Annunzio”, Chieti–Pescara, Italy (approval code SALI, N. 19 of the 10 September 2020) approved the collection and the use of saliva [27]. The healthy volunteers refrained from oral hygiene for 24 h, did not have any active oral caries, periodontitis, dental care in progress, or antibiotics therapy for at least three months prior to the beginning of the study.

Saliva was pooled, mixed, centrifuged (16.000× *g* for 1 h at 4 °C), and filtered from microorganisms by filters with pore diameters of 0.8, 0.45, and 0.2 μm. Saliva samples were considered sterile if no growth could be detected in both aerobic and anaerobic atmospheres after incubation for 24–48 h at 37 °C [27]. Sterile saliva was collected in sterile tubes and kept frozen until needed for the study.

### 2.3. Microbial Strain

*Streptococcus mutans* CH02, a clinical strain isolated from caries which was sourced from the private collection of the microbiology laboratory of the University “G. d’Annunzio”, Chieti–Pescara, was used in this experimental study [28]. The frozen (−80 °C) strain was recovered in Brain Heart Infusion broth (BHI, Oxoid, Milan, Italy) overnight at 37 °C under anaerobic condition. Then, the broth culture was diluted 1:10 in BHI broth (OXOID) containing 1% (*w*/*v*) sucrose and refreshed for 2 h at 37 °C in a shaking thermostatic water bath (160 rpm). Bacterial suspension was prepared using a spectrophotometer (Eppendorf, Milan, Italy) to obtain an optical density of OD_600_ = 0.12, corresponding to 9 × 10^6^ CFU/mL. This bacterial suspension was used for the experiment.

### 2.4. Experimental Design

All composite disk specimens were placed in 96-well polystyrene microtiter plates and sterilized through top and bottom surface exposure to ultraviolet UV light for 40 min [29]. Then, the sterile specimens were inoculated for 2 h in saliva at 37 °C in a shaking incubator with slight agitation to form the protein pellicle layer on the surface and to provide bacterial adhesion. Biofilms were grown on each composite disk, coated with saliva, by inoculation of 200 µL of standardized *S. mutans* CH02 bacterial suspension and incubation at 37 °C for 24 h under anaerobic condition, and then for another 24 h in aerobic atmosphere, in accordance with a previous study [28].

Negative controls, consisting of noninoculated composite disks, were also prepared. After incubation, the planktonic microbial growth was carefully removed from each well and analyzed for:

(i). Total mass amount by measuring the planktonic optical density (OD_600nm_);
(ii). Planktonic CFU count of the bacterial cells (CFU/mL);
(iii). Planktonic bacterial metabolic activity by MTT assay;
(iv). Planktonic bacterial viability assay by live/dead staining.

The disks were rinsed three times with phosphate-buffered saline (PBS) to remove unbound bacteria, and the bacterial load on each disk was assessed for:

(i). Adherent CFU count for the quantification of cultivable cells;
(ii). Adherent OD for biofilm biomass evaluation, using Hucker’s crystal violet staining method (OD_570nm_);
(iii). Adherent biofilm metabolic activity by MTT assay;
(iv). Extracellular polymeric substances (EPS) of the biofilm matrix by the Concanavalin A assay;
(v). Biofilm morphology by SEM evaluation.

Each evaluation was performed in triplicate for three independent experiments.

### 2.5. Planktonic Optical Density Detection

The effects of composite disks on the growth in the biofilm supernatant was determined. The planktonic phase, coming from the *S. mutans* CH02 biofilm formation, was removed from each well and transferred to wells of a new 96-well polystyrene flat-bottomed microtiter plates to evaluate the total mass amount by determining the OD_600nm_ with ELISA reader (SAFAS, Munich, Germany). For the detection, the planktonic phases coming from 39 disks (10 tests and 3 negative controls for each different CR material) in triplicate, for a total of 117 disks, were analyzed.

### 2.6. Planktonic CFU Count

To evaluate the ability of the different composite disks to influence the growth and viability of *S. mutans* CH02, the count of CFU/mL of planktonic bacteria was determined. The planktonic bacterial phase, removed from the wells containing the resin disks, was vortexed, diluted, and spread on Tryptic Soy Agar (TSA) plates and incubated for 24–48 h in anaerobic conditions at 37 °C. Then, the CFU/mL count was performed. For the detection, the planktonic phases coming from 30 disks (10 for each different CR material) in triplicate, for a total of 90 disks, were analyzed.

### 2.7. Planktonic MTT Assay

The planktonic growth was placed in a 96-well flat bottom microtiter plate and incubated with 20 μL of MTT (3-(4,5-dimethylthiazol-2-yl)-2,5-diphenyltetrazolium bromide) labeling reagent (Sigma–Aldrich Chemical Co., St. Louis, MO, USA) at concentration of 5 mg/mL for 2 h, as indicated by the manufacturer. After incubation, 100 μL of Dimethyl Sulfoxide (DMSO, Sigma–Aldrich Chemical Co., St. Louis, MO, USA) was added at each well and incubated for 10 min at the dark. Medium incubated with composite disks served as negative control. The assay is based on the metabolic conversion of water soluble MTT compound to a colored insoluble formazan derivate. Viable cells with active metabolism convert MTT into formazan; however, dead cells lose this ability [30]. The optical density reading was measured spectrophotometrically at 570 nm by ELISA reader (SAFAS, Munich, Germany). For the detection, the planktonic phases coming from 39 disks (10 tests and 3 negative controls for each different CR material) in triplicate, for a total of 117 disks, were analyzed.

### 2.8. Planktonic Bacterial Viability Assay

For the evaluation of planktonic cells viability, the cells were observed at fluorescent Leica 4000 DM microscope (Leica Microsystems, Milan, Italy). The planktonic bacterial phase was removed from each well and stained using the live/dead BacLight staining (Invitrogen, Milan, Italy), as previously reported [31]. The microscopic observation allowed the differentiation between live and dead cells based on the relative green and red fluorescence from SYTO 9 (500 nm) and propidium iodide (635 nm) staining. Ten fields of view, randomly chosen for each disk, were examined. Ten fields of view, randomly chosen for each planktonic phase coming from 9 disks (3 for each different CR material) in triplicate, for a total of 27 disks, were examined.

### 2.9. Adherent Bacteria CFU Count

The number of adhered viable bacteria on the surface of the specimens was determined to evaluate the ability of *S. mutans* to colonize the different composite disks, as previously described [32].

Briefly, after cultivating for 48 h, the adherent viable cells were washed with PBS to remove nonadherent cells. The disks were placed in a sterile test tube containing 1 mL PBS. Then, each test tube was placed in a 4 kHz ultrasonic water bath (Euronda, Italy) for 4 min followed by vortex mixing for 2 min to detach the bacteria adhering to the surface of each disk. The live/dead analysis was performed to confirm that all detached cells were viable and disaggregated. Then, serial 10-fold dilutions were carried out, plated on TSA plates, and incubated overnight at 37 °C, followed by counting of CFU/mL. For this detection, 30 disks (10 for each different CR material) were analyzed in triplicate, for a total of 90 disks.

### 2.10. Biomass Quantification by Optical Density (OD_570nm_)

After 48 h (biofilm formation), adherent viable biomass assessment was performed. A crystal violet (CV) staining was used to evaluate relative biofilm biomass formed by *S. mutans* CH02 on composite surfaces.

The disks were washed three times with PBS, fixed by air drying, stained with crystal violet 0.1% (Sigma–Aldrich, Milan, Italy) for 1 min and washed with PBS. After drying, bound CV was eluted with ethanol for reading. After 10 min, the composite disks were removed and the biofilm formation was quantified by measuring absorbance at 570 nm with a microplate reader (SAFAS, Munich, Germany). The absorbance of the eluted stain is proportional to the concentration of biofilm biomass formed on the sample surface [33]. For this detection, 39 disks (10 tests and 3 negative controls for each different CR material) were analyzed in triplicate, for a total of 117 disks.

### 2.11. Adherent Bacteria MTT Assay

To evaluate the metabolic activity of the biofilm formed by *S. mutans* CH02 on composite surfaces, the disks were incubated with 100 μL of MTT (3-(4,5-dimethylthiazol-2-yl)-2,5-diphenyltetrazolium bromide) labeling reagent (Sigma–Aldrich Chemical Co. St. Louis, MO, USA) at the concentration of 5 mg/mL for 2 h, as indicated by the manufacturer. A total of 100 µL of supernatant from each composite disk was transferred to a 96-well flat bottom microtiter plate, and the optical density reading of formazan derivative was read at 570 nm using an ELISA reader spectrophotometer (SAFAS, Munich, Germany). Disks incubated with media alone served as negative control. For this detection, 39 disks (10 tests and 3 negative controls for each different CR material) were analyzed in triplicate, for a total of 117 disks.

### 2.12. Concanavalin Assay

To analyze the extracellular polymeric substances (EPS) of the biofilms matrix, rhodamine-labeled Concanavalin A (rhodamine-conA) (Vector Laboratories, Burlingame, CA, USA), was used for its ability to bind to d−(+)−glucose and d−(+)−mannose groups on EPS.

The sessile bacterial population, adherent on composite disks, was washed with 1 mL of PBS twice, stained with 500 μL of the rhodamine-conA (10 μg/mL) solution, and incubated in the dark at room temperature for 30 min. Then, the excess staining solution was removed, and the stained specimens were rinsed with 1 mL of PBS and examined under fluorescence Leica 4000 DM microscope (Leica Microsystems, Milan, Italy). The fluorescence microscopy was used to obtain images using an excitation of 514 nm and an emission wavelength of 600 ± 50 nm. For this detection, 9 disks (3 for each different CR material) were analyzed in triplicate, for a total of 27 disks.

### 2.13. Scanning Electron Microscope (SEM) Analysis

After 48 h of in vitro biofilm formation, 5 specimens from each group were fixed for 1 h in 2.5% glutaraldehyde, dehydrated in six ethanol washes (10%, 25%, 50%, 75%, and 90% for 20 min and 100% for 1 h), and then dried overnight in a bacteriological incubator at 37 °C. Then, they were coated with gold (Emitech K550, Emitech Ltd., Ashford, Kent, UK) and observed carefully under a SEM (EVO 50 XVP LaB6, Carl Zeiss SMT Ltd., Cambridge, UK) at 15 kV, under 500×, 1000×, and 2000× magnifications. The representative micrographs of the biofilm on the specimens’ surface were recorded, and their descriptive analysis was performed.

### 2.14. Statistical Analysis

Means and standard deviations for data collected following the quantitative experiments (Planktonic OD_600nm_, Planktonic CFU count, Planktonic MTT, Adherent Bacteria CFU count, Biomass Quantification by OD_570nm_, Adherent Bacteria MTT) were calculated in each group. Statistical analysis was performed using SPSS for Windows version 21 (IBM SPSS Inc, Chicago, IL, USA), by means of the analysis of variance (ANOVA) and Tukey tests for posthoc intergroup comparisons. Homogeneity of variances and normality of the data sets were respectively confirmed by means of Levene’s and Kolmogorov–Smirnov tests. *p*-values less than 0.05 were considered significant.

## 3. Results

The results of the quantitative tests performed on the three composites examined in the study are summarized in Table 2.

### 3.1. Planktonic Optical Density Detection

The planktonic *S. mutans* OD_600_ mean values obtained in presence of VOCO, VD, and ES2 composite disks and control are shown in Table 2. In respect to the control, statistically significant (*p* < 0.05) OD_600_ values were recorded in presence of all tested composite disks. A major percentage of OD_600_ reduction in respect to the control was obtained in presence of VD; instead, a slightly decreased OD_600_ reduction was shown in presence of VOCO and ES2, with no statistically significant differences.

### 3.2. Planktonic CFU Count

As shown in Table 2, in presence of VOCO, the amount of CFU/mL was 5.8 × 10^7^ ± 5.3 × 10^7^ with a percentage of reduction in respect to the control of 69.47%. A similar trend was recorded in presence of VD and ES2 with 2.8 × 10^7^ ± 2.6 × 10^6^ CFU/mL and 2.7 × 10^7^ ± 1.4 × 10^6^ CFU/mL, respectively. In presence of VD and ES2, the percentage of CFU/mL reduction in respect to the control was about 85%. All CFU/mL results were significantly different in respect to the control (*p* < 0.05), and the CFU/mL obtained with VOCO were also significantly different in respect to the CFU/mL obtained with VD and ES2 (*p* < 0.05).

### 3.3. Planktonic MTT Assay

The planktonic metabolic activity showed the major value obtained with VD (0.549 ± 0.136) in respect to the VOCO (0.451 ± 0.094), ES2 (0.448 ± 0.085), and control (0.442 ± 0.120). In general, similar metabolic activity values were obtained in presence of all tested composite disks and control with no statistical significance (*p* > 0.05) (Table 2). All recorded cells were metabolically active.

### 3.4. Planktonic Bacterial Viability Assay

**Figure 1:**
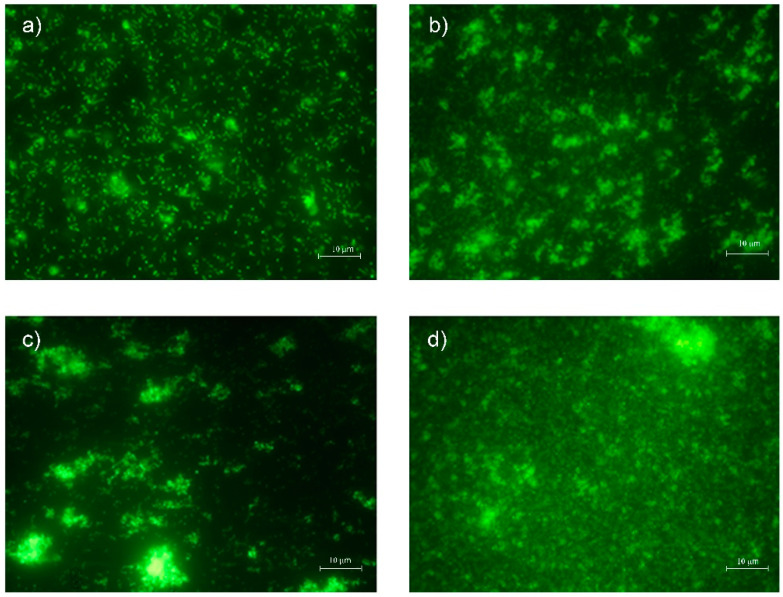
Representative live/dead images of *Streptococcus mutans in* planktonic phase of (**a**) control, (**b**) VOCO, (**c**) VD, and (**d**) ES2.

Typical live/dead images of planktonic *S. mutans* cells after 48 h of incubation on composite disks are shown in Figure 1. The live/dead images showed remarkable green viable cells in all conditions. In fact, no difference in terms of killing action was observed when VOCO, VD, and ES2 were compared to the control. A reduced number of cells was recorded in presence of the composite disks, when compared to the control.

### 3.5. Adherent Bacteria CFU Count

Similar numbers of CFU/mL adherent on VD and ES2 were obtained. The lowest CFU/mL value was recorded in presence of VOCO (1.5 × 10^5^ ± 2.9 × 10^5^), with statistically significant differences in respect to VD and ES2 (Table 2) (*p* < 0.05).

### 3.6. Biomass Quantification by Optical Density (OD_570nm_)

The best antibiofilm biomass effect was obtained with VOCO (0.4775 ± 0.1548), with a significant reduction in respect to ES2 (*p* < 0.05). A statistically significant difference between VD and ES2 (0.6364 ± 0.2376) was also recorded (Table 2), with VD showing a relevant increase in biomass biofilm compared to that of ES2.

### 3.7. Adherent Bacteria MTT Assay

Significant metabolic activity reductions were obtained in presence of all tested composite disks in respect to the control (*p* < 0.05) (Table 2). No difference between VOCO, VD, and ES2 disks was detected. Few cells were detected on the composite disks corresponding to the low metabolic activity in respect to the control.

### 3.8. Concanavalin Assay

**Figure 2:**
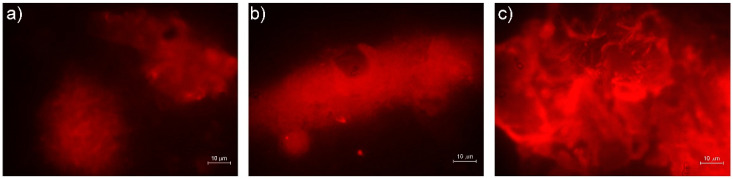
Concanavalin A assay. (**a**) VOCO shows a compact matrix, less than ES2, correlated with quantification of biomass produced by *Streptococcus mutans* biofilm; (**b**) VD shows a compact matrix, less than ES2, correlated with quantification of biomass produced by *Streptococcus mutans* biofilm; and (**c**) ES2 shows increased production of sugars from biofilm matrix.

The polysaccharides matrix production by *S. mutans* biofilms on different composites disks are plotted in Figure 2. A major production of carbohydrates was displayed on ES2 disks in respect to the other samples (c). As shown in Figure 2a,b, in presence of VOCO and VD a scarce EPS matrix was detected, with no significant differences.

### 3.9. SEM Analysis

**Figure 3:**
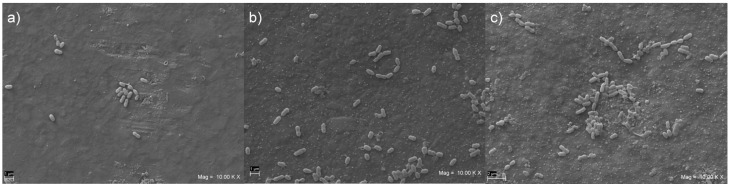
Representative SEM images (10.00 KX) of *Streptococcus mutans* biofilm formed on disk-shaped specimens from VOCO (**a**), VD (**b**), and ES2 (**c**) groups.

Representative SEM images of the *S. mutans* biofilm formation on the surface of the disk-shaped specimens are shown in Figure 3a–c. After 48 h of in vitro biofilm formation, the presence of the *S. mutans* cells was noted in all groups, but biofilm formation time was not sufficient to coat entirely the surface with *S. mutans* cells. Large adherent aggregates were observed in the ES2 group, which had the highest quantity of bacteria on its surface (Figure 3c), whereas small aggregates were found on VD e VOCO groups, with an apparently reduced biofilm formation (Figure 3a,b).

## 4. Discussion

In this study, the behavior of *Streptococcus mutans* exposed to three commercially available, nanohybrid, dental CR-based materials, coated with human saliva, was quantitatively assessed through the evaluation of total microbial population, the viable count, and the metabolic activity in planktonic and sessile growth mode. To better analyze the potential influence of different chemical formulations on biofilm formation, all samples were subjected to the same standardized light-curing protocol through two glass slides and inside polyvinylsiloxane molds, to predictably achieve the smoothest surface possible with the least amount of surface roughness. This allowed to remove the effects of a potentially confounding variable, the surface topography, which is also hypothetically able to affect biofilm adhesion [34,35]. Based on the achieved results, the null-hypothesis tested had to be rejected: statistically significant differences were observed when comparing the antibacterial and antibiofilm properties of chemically different CR materials.

Focusing on Planktonic Optical Density assay, a statistically significant reduction emerged in planktonic cells exposed to all the nanohybrid CRs, compared to that of the control. Kim et al. showed that bis-GMA inhibited the planktonic growth of *S. mutans* in media containing glucose, fructose, or mannose [36]. However, since in our research bis-GMA was present just in two out of the three tested resins, it is possible to suppose that the *S. mutans* decrease can be determined even by other monomers, or their combination.

The Planktonic Optical Density reduction was in accordance with the results of the planktonic CFU assay, which showed for all resins a statistically significant reduced planktonic CFU count compared to that of the control. Furthermore, the planktonic microbial cell populations detected by CFU count, when studied for their viability, displayed a green color suggesting a bacteriostatic effect for all composites. Such behavior was more relevant for ES2 and VD supposing a major capability of *S. mutans* to adhere to these materials with a reduction in planktonic counterpart, which is in line with the findings of the Adherent Bacteria CFU count test.

Regarding the Adherent Bacteria count, a significant increase was observed for ES2 and VD compared to that of VOCO. Indeed, for VOCO, results showed a significant anti-adhesive effect with a reduction in Adherent CFU count, biomass quantification, and presence of EPS matrix. This material expressed the best performances compared to that of the other composites with significance in terms of antiadhesive action and a general reduction in biomass quantification and MTT detection. Of course, antiadhesive and antibacterial properties should be cautiously pondered, taking into account also any potential cytotoxicity due to the monomers included in (and released from) adhesive dental materials [37,38]. Several in vitro studies demonstrated that the potential cytotoxicity of the CR organic components is mainly due to the residues of free methacrylate monomers following the phase of polymerization, which may trigger the production of prostaglandin E2 (PGE2), the expression of cyclooxygenase 2 (COX2), and a proinflammatory activation through the increase in interleukin-1β (IL-1β), IL-6, and nitric oxide (NO) [39,40]. The capability of resin monomers to influence cellular physiology and adaptive cell responses by increasing ROS production was also reported [41,42]. In this regard, a recent study showed reduced cytotoxic and genotoxic effects for VOCO, compared to that of VD, toward human gingival fibroblasts, somehow strengthening the clinical relevance of the present findings [43].

A lower biomass production of biofilms and polysaccharides results in thinner biofilm thickness, lower biofilm matrix barrier for protection, and less carbohydrate amount accumulation. This could be clinically beneficial, as the resulting biofilm would be more susceptible to the buffering action of saliva, fluoride ions, and antibacterial agents.

Interestingly, the MTT assay on adherent cells led to concordant results: metabolic activity underwent a significant reduction following the exposure to all the tested composites, but it appeared relatively and slightly increased in presence of ES2 and VD, compared to that of VOCO. As observed by Aqawi et al., the metabolic activity-related reduction in preformed biofilm could be associated with the modification of membrane polarization [44].

As already reported by Kim et al., cells grown under bis-GMA showed significantly increased surface hydrophobicity, which could potentially enhance the ability of *S. mutans* to adhere to hydrophobic surfaces [36]. However, in the present study, both bis-GMA free (VD)- and bis-GMA-based (ES2) composites led to a significant increase in adherent *S. mutans* CFU count, compared to that of a third bis-GMA based (VOCO) material. Thus, as already pointed out concerning the Planktonic Optical Density results, also when dealing with the adherent CFU count, all the composite components might simultaneously interact; thus, bis-GMA should not be seen as the only monomer able to modulate the adherent properties of bacteria. Further studies would be needed to clarify such effects properly and comprehensively, with respect to the complex formulation of commercial CR-based materials. Also, the filler amount/size/shape might represent another possible factor influencing bacterial adherence to resin composite surface, as suggested by previous papers [21,22,45]. VOCO had a higher proportion of filler particles than ES2. VOCO contained approximately 89 wt% of filler particles, while ES2 contained 78 wt%. From this point of view, the present results seem in line with Ikeda et al., who observed resin composites with a higher filler content showing a reduced biofilm retention [20,46].

The presence of bacterial biofilm, in association with a gap at the margin between the composite and the tooth structure, may lead to the development of secondary caries [47]. This is, in fact, one of the main reasons for restorations replacement [48]. For this reason, the present research focused on the adhesive and biofilm-forming capabilities of *S. mutans* toward CRs with different chemical compositions, as this microorganism can be isolated in almost all carious lesions [10]. *S. mutans* tends to accumulate more on composites than on enamel or other restorations [49], and its presence on tooth surfaces is usually followed by caries after 6 to 24 months [50]. Then, the inhibition of *S. mutans* biofilm formation is a key goal for preventing dental caries.

Based on the present results, VOCO showed a strong reduction in biofilm growth in terms of CFU count, biofilm biomass, metabolic activity, and polysaccharide production compared to those of VD and ES2. These features could indicate VOCO as a more promising restorative material, even though it also allowed microorganism growth to a certain extent. The limitation of this study is related to the use of a single clinical strain of *S. mutans* and a static model. The interesting results herein obtained suggest carrying out further studies, including a multispecies dynamic biofilm model, to better evaluate the susceptibility of adhesion of microorganisms to resin-based materials.

## Figures and Tables

**Table 1:** Composite resins included in the study.

Group	Material	Manufacturer	Lot Number	Composition
VOCO	GrandioSO Shade A2 (Nanohybrid)	Voco GmbH (Cuxhaven, Germany)	1847313	89% (*w*/*w*) fillers (1 μm glass ceramic filler, 20–40 nm silicon dioxide fillers), Bis-GMA *, Bis-EMA *, TEGDMA *.
VD	Venus DiamondShade A2 (Nanohybrid)	Kulzer GmbH (Hanau, Germany)	K010070	80–82% (*w*/*w*) fillers (5 nm–20 μm barium aluminum fluoride glass fillers), TCD-UA *, UDMA *, TEGDMA *.
ES-2	Clearfil Majesty ES-2 Classic Shade A2 (Nanohybrid)	Kuraray (Chiyoda, Tokyo, Japan)	7D008	78% (*w*/*w*) fillers (0.37 μm–1.5 μm silanated barium glass fille, pre-polymerized organic fillers), Bis-GMA *, hydrophobic aromatic dimethacrylate.

* Bis-GMA = Bisphenol A-glycidyl methacrylate; Bis-EMA = Bisphenol A-glycidyl methacrylate ethoxylated; TEGDMA = Triethylene glycol dimethacrylate; TCD-UA = Tricyclodecane-urethane acrylate; UDMA = Urethane Dimethacrylate.

**Table 2:** *Streptococcus mutans* detection on three resin composites investigated.

Streptococcus Mutans CH02	VOCO	VD	ES2	CTRL+
Planktonic OD_600nm_ (SD)	0.3054 ^b^	0.2931 ^b^	0.3117 ^b^	0.3978 ^a^
	(0.0567)	(0.0540)	(0.0532)	(0.0491)
Planktonic CFU count (×10^5^ CFU/mL) (SD)	583.0 ^b^	281.0 ^c^	273.0 ^c^	1900.0 ^a^
	(53.4)	(26.4)	(14.1)	(353.3)
Planktonic MTT (SD)	0.451 ^a^	0.549 ^a^	0.448 ^a^	0.442 ^a^
	(0.094)	(0.136)	(0.085)	(0.120)
Adherent Bacteria CFU count (×10^3^ CFU) (SD)	150.0 ^b^	252.0 ^a^	248.3 ^a^	
	(29.4)	(39.7)	(47.8)	
Biomass Quantification OD_570nm_ (SD)	0.4775 ^b^	0.6364 ^b^	1.6040 ^a^	
	(0.1548)	(0.2376)	(0.2075)	
Adherent Bacteria MTT (SD)	0.003 ^b^	0.040 ^b^	0.035 ^b^	0.367 ^a^
	(0.004)	(0.013)	(0.021)	(0.274)

VOCO, VD and ES2 are the experimental groups, based on the materials described in Table 1. CTRL+ indicates the positive control group. Same superscript letters indicate not statistically significant differences.

## Data Availability

The data presented in this study are available on request from the corresponding author.
